# The role of drinking in new and existing friendships across high school settings^*^

**DOI:** 10.4236/health.2013.56A3004

**Published:** 2013-06-20

**Authors:** Jacob E. Cheadle, Deadric Williams

**Affiliations:** The University of Nebraska-Lincoln, Lincoln, America

**Keywords:** Adolescent, Alcohol Use, Drinking, Teen, Social Network, Friends, Friendship

## Abstract

We use 9 Add Health high schools with longitudinal network data to assess whether adolescent drinkers choose friends who drink, prefer friends whose friends drink, if selection differs between new and existing friendships, and between schools. Utilizing dynamic social network models that control for friend influences on individual alcohol use, the results show that drinkers do not strongly prefer friends who drink. Instead, they favor close friends whose friends’ drink, suggesting that alcohol matters for selection on the social groups and environments that friends connect each other to. The role of alcohol use differs by whether friendships are new or existing, however, with bridging connections being less stable. Moreover, selection processes, and the implications of alcohol use for friendship, vary in important ways between schools.

## 1. INTRODUCTION

The prevalence of alcohol use in adolescence suggests that it is an important avenue by which teens integrate socially with peers away from adult supervision. By 12th grade over 70% of teens have tried alcohol and low noncontinuation rates below 10% indicate that most adolescents continue to drink [1]. The high rates of individual use reflect the fact that teens regularly expose each other to alcohol. Approximately 50% of 12th graders report they often drink in the company of other youth and that most or all of their friends drink frequently, and 75% report that one or more friends drink until drunk each week [2]. As a common, socially embedded activity in adolescence, alcohol use taps into a core set of social behaviors not condoned by adults but that are key to understanding adolescent society [3]. The purpose of this study, therefore, is to examine the role of alcohol use in the friendship formation (creation of a new friendship) and durability (maintenance of an existing friendship) during a developmentally important stage of life. We address this purpose by applying longitudinal stochastic actor based models of social network dynamics [4] to 9 schools from the National Longitudinal Study of Adolescent Health (Add Health).

## 2. LITERATURE REVIEW

Social integration refers to the number of social relations one has, in addition to the type and frequency of contact [5]. We continue the traditional focus on social networks as indicators of integration [6], but take a dynamic view that considers friendship change and stability in peer relationships. We emphasize the role of homophily, the tendency for individuals with similar characteristics to befriend one another, as one important avenue by which alcohol use can promote social relationships that foster social integration.

Researchers have long noted that friendships tend to be homophilous on social characteristics [7]. This tendency is replicated for adolescent health-risk behaviors [8] and alcohol use is no exception [9]. Given the wide-spread diffusion of drinking and its associated risks [10] over this phase of life, there is a critical need for research articulating how alcohol use is intertwined in friendships as a socially integrative behavior. Recent findings generally support dual roles of alcohol use in homophilous friendship selection and social influence, though there is disagreement about when each process emerges over adolescence [11–13]. We focus here on the role of homophilous friend selection. Thus, our first hypothesis is dyadic: *adolescents prefer to be friends with teens who have similar alcohol use levels*.

Adolescent socializing takes place both with close friends and broader collections of peers, some of whom are sociometrially closer than others. For example, teens often spend time in the company of friends’ friends [14], which leads to indirect connections, shared social environments, and new friendship opportunities. Reflecting this complexity, recent studies report that friends’ friends influence health risk behaviors [15,16] including alcohol use [17]. This points to the need to consider interpersonal processes extending beyond immediate friends and out to less intimate acquaintances [18,19].

Payne and Cornwell [19] argue that adolescents are socially strategic and that they adopt behaviors to impress their close friends by modeling the actions of indirect but still closely connected contacts. However, this logic may also work the other way: adolescents who enjoy drinking and partying may prefer friends who connect them to other drinkers, with the result that they form friendships strategically. To the extent this is so, friendships may form precisely because of the broader set of connections they bring when a party behavior like alcohol use that takes place in social contexts is considered. Drinking may thus integrate adolescents beyond their closest social ties and out into broader social circles—effectively expanding the social milieu they participate in, and making peers who connect them to other drinkers more attractive. Consequently, our second hypothesis is that *adolescent drinkers prefer friends whose friends drink*.

We further suggest that alcohol use should promote durable bonds if it is socially integrative. Friendships, however, change all the time [20]. The question of whether alcohol use plays different roles as friendships develop and mature is a possibility that prior research has only partly addressed. For example, using tabular methods, Fisher and Bauman [21], found that alcohol use was more strongly related to the acquisition of friends, was less strongly related to influence, and was not related to friendship durability. If alcohol use is associated with higher turnover among friends, however, then drinking undercuts integration even while setting the stage for it. Asymmetries in friendship processes between new and existing friendships would be reflected in differential tendencies for relations to form versus being maintained over time.

Thus, our final hypotheses modify our first two by disaggregating friendship into *formation* and *durability* processes. Prior work on social network selection using statistical models has conflated these processes. With this in mind, the third hypothesis is that *alcohol users form new friendships with one another* and the fourth hypothesis is that *relationships homophilous on alcohol use are less durable* and thus are less likely to be maintained over time.

## 3. DATA AND METHODS

Data come from Add Health’s in-school assessment at wave 1 (observation 1) along with its in-home wave 1 and 2 (observations 2 and 3) components. Add Health is a cluster stratified study of 7th–12th grade youth begun in 1994 with in-school questionnaires administered to approximately 90,000 students in 140 schools. A nationally representative sample of 20,000 students was drawn from the in-school study, and data were collected in-home in 1995 and again approximately one year later at wave 2. This longitudinal sample consisted of a special subsample of 16 “saturated” schools in which friendship data were collected for all attending 7–12th grade students. The sample for this study utilizes 9 of the high schools (N = 3329) with response rates adequate for social network analysis [22]. 1,704 adolescents come from the large, racially heterogeneous high school, 829 from a middle-sized predominantly white high school, and 798 from the seven small (N < 300) k-12th grade high schools. Missing network data were handled using the composition change method [23], so that all youth were included in the analysis and allowed to enter the study later or leave early (e.g., graduates, movers, dropouts). Missing alcohol and attribute data were treated as noninformative and imputed within the model [22].

### 3.1. Variables

The focal dependent variable, the friendship network matrix, maps the interconnections between individuals and so captures the system and structure of relationships among adolescents in the 9 schools (see [24]). The adolescent friendship network is constructed from two sets of variables requesting nominations of up to five male and five female friends from the school roster at each observation. The repeated, longitudinal assessments of the social network provide the analytic leverage for studying friend selection and thus social integration. Due to a sampling error a subset of students was given only a single nomination opportunity at observation 2 (the wave-1 in home assessment) so that the full friendship network was not captured for approximately 40% of the small school youth, and 5% of those attending the medium white and large minority schools. We randomly carried either the observation 1 or 3 nominations forward or backwards in order to backfill the missing nominations and preserve the network. Descriptive statistics for the network are provided in Table 1.

Alcohol use frequency, the focal independent variable, is based on the question, “During the last 12 months, on how many days did you drink alcohol?” This item employs a standard alcohol use intensity assessment that was measured as a six-point scale with values for never drinks, once or twice in the last year, once a month or less, 2 3 days a month, 1 2 days a week, and 3 to 5 days a week, and every day or almost every day. Because we found that sparse distributions in the upper categories produced instability in the estimation routines when peer influence was controlled for, we top-coded alcohol use at the fourth category—2 3 days per month. Descriptive statistics for alcohol use similarity among direct and indirect friends are shown in Table 1, and statistics describing individual alcohol use over time are shown in Table 2.

An indicator for *female* is included to reflect the gendered structure of adolescent social networks. Race/ethnic background is included in two ways to reflect compositional differences across schools. First, an indicator of *nonwhite* status for all schools except the large minority school, while indicators for *Hispanic*, *Asian*, and *white* are included with *African American* omitted in this school. *Parent education* is included as a linear 5-category background control. Additional factors related to alcohol use include the frequency with which the responding *parent drinks alcohol* (1 = never to 6 = nearly every day), an indicator for *whether alcohol is easy to get*, whether the youth is a *regular smoker* is a time-varying covariate, an indicator for whether or not the adolescent’s observation 2 nominations were accidenttally restricted to only a single friend (as noted above), and the number of off-list nominations (also time-varying).

### 3.2. Analytic Strategy & Analysis Plan

The analysis employs the new class of Simulation Investigation for Empirical Network Analysis (SIENA) models developed by Snijders [25] and colleagues (e.g., [4]). The SIENA approach models changes in the friendship network as a function of individual, dyadic, and extra-dyadic alcohol use. Observation 1 is a starting point for the estimation routine so that parameters capture changes in the network across observation periods.

Coefficient estimates capture changes in network statistics between observations and an agent based simulation model is utilized to update the parameters and estimate their uncertainties. The SIENA model is unique in that it can be used to jointly model changes in the friendship matrix (selection) and individual behaviors (influence) so that each process can be isolated from the other. Moreover, a number of structural network characteristics can be controlled for with this approach (we include terms for out degree, reciprocity, transitive triplets, and 3-cycle). Focal coefficients are presented in terms of *ego* (nominations of friends), *alter* (nominations by others), and their interaction.

The school-specific networks for the 7 smaller k-12th schools were grouped into a single matrix and analyzed jointly as others have done with these schools [26,27]. The small schools, medium-sized mostly white and the large urban minority schools were then analyzed separately using the RSIENA software. Differences in key parameters were then compared across schools using t-tests.

## 4. RESULTS

Logit coefficients for the alcohol use selection parameters are shown along with standard errors in Table 3 for each of the small (SS), medium (MS), and large school (LS) networks. Model 1 shows results for the baseline alcohol selection model including controls for reciprocity, off list nominations, and whether or not the respondent was in the restricted nomination sample at observation 2. As with most studies conducted utilizing the SIENA approach, the homophilous ego-alter selection parameter is held equal for the formation of new friendships and the durability of existing relationships. Although the effect magnitudes are consistent across networks, Model 2 suggests that this approach masks heterogeneity in both alcohol-based selection and the differential contributions of alcohol use to new and existing friend processes across networks. For both the SS and MS, alcohol use is related to new friendships, and the effect is much larger than in Model 1 because the effect of homophily on friendship durability in existing relationships is close to 0. The ego-alter creation coefficient is also significant for the LS, but in contrast to the others, friendships among drinkers are significantly *less* durable—in this model, alcohol use is found to create opportunities for social integration through the creation of new friendships, but also to undercut it by facilitating their turnover.

Model 3 further elaborates the role of drinking in friend selection processes by including the interaction between ego’s alcohol use with the average drinking of the potential/existing friend’s friends. The ego-alter creation effect reduces substantially across networks, even becoming non-significant for the MS. At the same time, indirect drinking selection is related to the creation of new friendships and their subsequent loss across networks. Moreover, the coefficient sizes are all substantially larger than for dyadic selection. The actual role of alcohol use in selection is somewhat more complicated than presented in Model 3, as shown in Model 4 when friend influence on alcohol use (of both close friends and their friends) is incorporated into the equation. When indirect connections to other drinkers are considered, dyadic selection is no longer found to be significant for the LS, though this reflects a large increase in the SE relative to the coefficient. Additionally, the loss of bridging ties to other drinkers is no longer significant in the LS, where indirect selection is found to primarily influence new friendships to other drinkers. In the SS and MS, however, existing dyadic friendships are more stable while the ego-alter creation parameter is no longer significant. The complex relationship between individuals and their friend’s friends’ alcohol use persist for these schools, however.

Model 5 adds the structural network parameters for transitive triplets and 3-cycle to account for structural processes that influence friendships [28]. These results suggest that durability of friendships bridging to other drinkers in the MS actually reflects network closure. In other words, homophilous friendships are more durable because they are embedded in larger sets of overlapping friendships. Model 6 adds the additional control variables (full results available upon request). In addition, although results changed between Models 3 and 4 when friend and friends’ friends’ influences on alcohol use were included, Model 6 and selection-only results (not shown) are quite similar, but the precision with which the parameters are estimated differs markedly. t-test results comparing coefficients across networks suggest somewhat different processes across settings. Although the processes are similar overall in the SS and MS, the LS differs from these schools: homophilous direct relationships are less durable in the LS (*t_MS_* = 3.14, *alter*, *ego × alter* keep friends *t_SS_* = 2.36 and *t_MS_* = 2.21), but bridging connections (*ego × alter at distance* = 2, *keep*) linking drinkers are less durable in the SS (*t* = 12.32) and MS (*t* = 12.20).

## 5. DISCUSSION

Understanding alcohol use preferences in friendship choices is integral for deepening theoretical understanding of an important social process, social integration, during adolescence. One mechanism of social integration, homophilous friendship selection, has been a primary concern among researchers because it is generally not accounted for in peer influence estimates and the conesquences can be quite diverse: biased expectations for interventions (e.g., [29]), obscured potential intervention strategies [30], and adversely modified intervention effects in the field [31]. Given the dangers of friend selection for undermining programs, and the fact that studies suggest that selection processes typically bias and even trump peer influences in importance [21], understanding it substantively is as critical as controlling for it in influence studies.

Although a number of recent studies now address friend influences on alcohol use accounting for homophilous selection [11–13], we argued that friendship selection processes are more complicated than recent studies using longitudinal network models have indicated [21]. Using dynamic social network analysis, we have in fact showed that to be the case. First, in contrast to most prior studies, we approached friendship selection from the view that it comprises both creation and durability processes that could differ from one another. The findings from 9 schools of varying sizes and composition support this contention. Approaching alcohol use from the perspective that its role in new and existing friendships is equivalent clearly masks heterogeneity in the role it plays in fostering friendships.

Second, drinking selection appears to be less a property of dyads, as has typically been assumed, and more about indirect selection that promotes access to other drinkers. Adolescent drinkers prefer friends that connect them to other drinkers, which suggests that friendship in adolescence has much to do with the social environments that adolescents provide one another through their social contacts. This was found to promote the creation of new friendships across networks, but these friendships were simultaneously found to be less stable. Network and other selection processes contribute to creating stability in these bridging connections, but they still tend to turnover more quickly. The result is that alcohol use is a pathway by which adolescents connect with one another, but it tends to create new ties at the expense of the old. Despite the small dyadic effect on friendship durability in the mid-sized school, the overall trend is that alcohol use sets the stage for integration but does not create deeper forms of social connection. Selection on other factors is necessary to create deeper forms of integration.

Our third point is that these processes vary across settings in important ways that are not yet fully understood: in some cases, alcohol use is associated with the loss of ties that bridge drinkers, but this is not always the case, as shown by the large minority school. Interestingly, differences across schools would not have been evident had we merely looked at the ego-alter interaction parameter, which is a baseline measure of homophilous selection. This effect was quite similar across schools, and differrences only emerged when we disaggregated the parameter for the creation of new and durability of existing ties. In essence, the conflicting role of alcohol in these processes drives the parameter towards zero and thereby lessens variations across networks.

This latter point also reveals important limitations specific to this study. The Add Health longitudinal net-work sample is one of convenience and it is idiosyncratic for that reason. At the same time, however, the schools are quite variant. Given that school size, composition, and urbanicity are all confounded in this study, future work with more diverse samples will be necessary to further illuminate heterogeneity in these processes.

The analyses are also limited in other ways. For ex-ample, the network is based on close friends. For this reason, it is important to recognize that indirect ties might very well be friends too and that the effects presented here reflect changes in the relative status of otherwise close groups of adolescents. If this is the case, it means that social network scholars may have often viewed the social network in terms that are too narrow and missed important aspects of adolescents’ social connections. Moreover, the study comprises a relatively narrow window of time, albeit one that captures a critical period when adolescents begin drinking and expanding their social milieu as they separate from their parents and form their own identities [32].

## Figures and Tables

**Table 1:** Descriptive network statistics for the total and school-specific samples.

Variable/Statistic	Total Sample			Small (SS)	Medium (MS)	Large (LS)

	Obs	Mean	Std. Dev.	N = 796	N = 829	N = 1704
Alcohol similarity (range:0 – 1)				0.64	0.55	0.61
Alcohol similarity at distance =2				0.72	0.73	0.74
Restricted nomination sample, obs. 2	3329	0.13	0.34	0.40	0.06	0.04
Off list nominations, obs. 1	3329	1.28	2.22	1.42	0.73	1.48
Off list nominations, obs. 2	3329	2.38	2.10	2.61	1.81	2.54
Off list nominations, obs.3	3329	1.81	2.18	2.51	1.47	1.65
Degree, obs.1				3.80	5.29	2.95
Degree, obs.2				3.21	4.27	2.17
Degree, obs.3				2.69	3.49	1.76
Jaccard distance, obs.1→2				0.51	0.38	0.37
Jaccard distance, obs.2→3				0.43	0.36	0.37

**Table 2:** Descriptive statistics for the total and school-specific samples.

Variable/Statistic	Total Sample			Small (SS)	Medium (MS)	Large (LS)

	Obs	Mean	Std. Dev.	N = 796	N = 829	N = 1704
Alcohol use, obs.1	2361	2.05	1.15	1.82	2.48	1.94
Alcohol use, obs.2	3325	2.05	1.18	1.79	2.42	1.98
Alcohol use, obs.3	2413	2.00	1.22	1.72	2.46	1.90
Female	3329	0.48		0.50	0.47	0.48
Grade	3292	10.44	1.29	9.47	10.33	10.94
Parent education	2656	2.55	1.10	2.63	2.66	2.44
Non-white	3326	0.54		0.15	0.06	0.95
White	1701	0.06				0.06
African American	1701	0.23				0.23
Hispanic	1701	0.39				0.39
Asian	1701	0.32	0.46			0.32
Regular smoker, obs.1	2365	0.12	0.33	0.14	0.23	0.06
Regular smoker, obs.2	3326	0.23	0.42	0.25	0.36	0.15
Alcohol is easy to get	3310	0.28		0.20	0.32	0.29
Parent drinks alcohol	2649	1.78	1.05	1.65	2.18	1.63

**Table 3:** Logit coefficients and standard errors across the model series for the alcohol friend selection parameters by school network.

Parameter	Model 1	Model 2	Model 3		Model 4		Model 5	Model 6	
*SS: Small Schools* (*N* = 796)									
Alter	−0.049*	−0.047*	−0.048*		−0.044		−0.018	0.035	
	[0.020]	[0.021]	[0.022]		[0.042]		[0.034]	[0.047]	
Ego	−0.123*	−0.125*	−0.172*		−0.291	+	−0.24*	−0.192	Ψ
	[0.025]	[0.026]	[0.043]		[0.149]		[0.117]	[0.186]	
Ego × alter	0.094*								
	[0.019]								
New friends: ego × alter		0.255*	0.107*		0.106		0.079	0.055	
		[0.035]	[0.036]		[0.086]		[0.090]	[0.128]	
Keep friends: ego × alter		−0.037	0.062		0.232*		0.232*	0.214	
		[0.033]	[0.041]		[0.100]		[0.098]	[0.134]	
New friends: ego × alter @ dist. =2			0.826*		1.001*		1.117*	1.007	Ψ
			[0.153]		[0.359]		[0.341]	[0.692]	
Keep friends: ego × alter @ dist. =2			−0.804*		−0.984*		−1.197*	−1.187*	
			[0.193]		[0.319]		[0.254]	[0.498]	
*MS: Medium School* (*N* = 829)									
Alter	0.042*	0.042*	0.041*		0.055*		0.034	0.058*	
	[0.012]	[0.012]	[0.012]		[0.025]		[0.022]	[0.026]	
Ego	−0.01	−0.01	−0.028*		−0.056		−0.057*	−0.047	Ψ
	[0.012]	[0.013]	[0.014]		[0.035]		[0.021]	[0.049]	
Ego × alter	0.083*								
	[0.010]								
New friends: ego × alter		0.197*	0.024		−0.03		−0.01	−0.028	
		[0.020]	[0.021]		[0.044]		[0.047]	[0.052]	
Keep friends: ego × alter		−0.027	0.075*		0.126*		0.07	0.062	Ψ
		[0.022]	[0.024]		[0.042]		[0.059]	[0.066]	
New friends: ego × alter @ dist. =2			0.834*		1.155*		1.198*	1.139*	
			[0.067]		[0.340]		[0.222]	[0.412]	
Keep friends: ego × alter @ dist. =2			−0.585*		−0.417*		−0.603*	−0.521*	
			[0.065]		[0.178]		[0.146]	[0.177]	
*LS: Large School* (*N* = 1704)									
Alter	−0.049*	−0.051*	−0.058*		−0.131*		−0.12*	−0.069*	
	[0.014]	[0.015]	[0.017]		[0.028]		[0.060]	[0.031]	
Ego	−0.025	−0.022	−0.061*		−0.14	+	−0.123	−0.103	Ψ
	[0.020]	[0.019]	[0.023]		[0.075]		[0.210]	[0.059]	
Ego × alter	0.073*								
	[0.014]								
New friends: ego × alter		0.261*	0.125*		0.138		0.174	0.162	Ψ
		[0.024]	[0.025]		[0.086]		[0.209]	[0.091]	
Keep friends: ego × alter		−0.117*	−0.053	+	−0.019		−0.096	−0.128*	
		[0.031]	[0.032]		[0.054]		[0.070]	[0.055]	
New friends: ego × alter @ dist. =2			0.719*		0.785*		0.682	0.589*	
			[0.075]		[0.266]		[0.987]	[0.183]	
Keep friends: ego × alter @ dist. =2			−0.679*		−0.179		−0.014	0.046	
			[0.085]		[0.175]		[0.754]	[0.187]	

p < 0.05;

Ψ not significant but p < 0.05 in the final model when influence is not adjusted for and where difference is due to precision of estimate.
